# Twenty-eight-day mortality in lung cancer patients with metastasis who initiated mechanical ventilation in the emergency department

**DOI:** 10.1038/s41598-019-39671-8

**Published:** 2019-03-20

**Authors:** Sun Hye Shin, Hyun Lee, Hyung Koo Kang, Joo Hyun Park

**Affiliations:** 1 Division of Pulmonology and Critical Care Medicine, Department of Medicine, Sungkyunkwan University School of Medicine, Seoul, Korea; 2 Division of Pulmonary Medicine and Allergy, Department of Internal Medicine, Hanyang University, School of Medicine, Seoul, Korea; 3 Division of Pulmonary and Critical Care Medicine, Department of Internal Medicine, Ilsan Paik Hospital, Inje University College of Medicine, Goyang, Korea; 4 Department of Emergency Medicine, Samsung Medical Center, Sungkyunkwan University School of Medicine, Seoul, Korea; 5 Department of respiratory medicine, College of Medicine, Kangwon National University, Chuncheon, Gangwon Korea

## Abstract

Few data are available regarding treatment outcomes in lung cancer patients with metastasis who initiated mechanical ventilation in the emergency department (ED). We aimed to evaluate 28-day mortality in lung cancer patients with metastasis who initiated mechanical ventilation in the ED. Patients with solid malignancy who initiated mechanical ventilation in the ED of a tertiary hospital were retrospectively identified and stratified into four groups according to the presence of lung cancer and metastasis. Among 212 included patients, the mortality rates by the 28^th^ hospital day were as follows: 44.2% (19/43) in non-lung cancer patients without metastasis, 63.2% (43/68) in non-lung cancer patients with metastasis, 52.4% (11/21) in lung cancer patients without metastasis, and 66.2% (53/80) in lung cancer patients with metastasis. In multivariable analysis, lung cancer patients with metastasis had significantly higher odds ratio for 28-day mortality than non-lung cancer patients without metastasis (adjusted odds ratio [OR] = 7.17, 95% confidence interval [CI] = 2.14–24.01). Sepsis-related respiratory failure (adjusted OR = 2.60, 95% CI = 1.16–5.84) and cardiopulmonary resuscitation (adjusted OR = 13.34, 95% CI = 4.45–39.95) over respiratory failure without sepsis and acute organ dysfunction process measured by sequential organ failure assessment (SOFA) score (adjusted OR = 1.15, 95% CI = 1.05–12.6) were independently associated with an increase in mortality rate. In conclusion, the treatment outcomes in lung cancer patients with metastasis who initiated mechanical ventilation in the ED were poor. Aggressive resuscitation versus end-of-life care in advance of an unexpected medical crisis should be considered in lung cancer patients with metastasis via a multidisciplinary approach with a consideration of underlying comorbid illnesses in the acute organ dysfunction processes.

## Introduction

Lung cancer is one of the most common solid cancers, and often requires medical intensive care unit (ICU) admission due to acute illness^1,2^. A previous study revealed that approximately 16% of all cancer-related ICU admissions are associated with lung cancer^3^. In addition, stage IIIB and stage IV lung cancer have shown annual increase of 6.6% in ICU use in the United States during the 1990s^4^. The prognosis of lung cancer is one of the worst among all cancers, and there is controversy regarding the usefulness of ICU care for this patient group.

Recently, it has been reported that the presence of lung cancer and emergency hospital admissions are particularly associated with higher mortality in patients with solid cancers who are admitted to the ICU^5,6^. In addition, among lung cancer patients admitted to the ICU, the need for mechanical ventilation and the advanced refractory cancer status were clinical factors predicting hospital mortality^2,7–11^. Considering the results of previous studies, the treatment outcomes in patients with far-advanced lung cancer (i.e., with distant metastasis) who initiated mechanical ventilation in the emergency department (ED) prior to ICU admission are expected to be even worse. Thus, the decision to triage these patients to receive mechanical ventilation in the ED should be made carefully. However, there have been few studies evaluating the treatment outcomes in patients with far-advanced lung cancer who initiated mechanical ventilation in the ED. Thus, we aimed to investigate the 28-day mortality in lung cancer patients with metastatic disease who initiated mechanical ventilation in the ED.

## Results

### Patients

**Figure 1:**
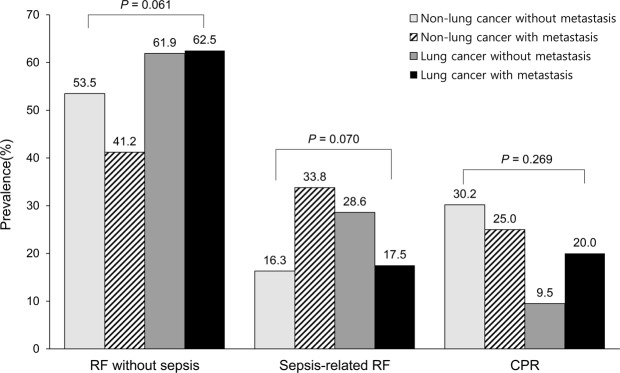
Indications for mechanical ventilation according to presence of lung cancer and metastasis. RF, respiratory failure; CPR, cardiopulmonary resuscitation.

Baseline characteristics of 212 cancer patients who were intubated for mechanical ventilation in the ED are summarized in Table 1. Patients were stratified into four groups as follows: 43 non-lung cancer patients without metastasis (20.3%), 68 non-lung cancer patients with metastasis (32.1%), 21 lung cancer patients without metastasis (9.9%), and 80 lung cancer patients with metastasis (37.7%). There were no significant differences in the clinical factors including age, body mass index, comorbidities except for chronic liver disease, and performance status. The proportion of male was higher in lung cancer patients compared to non-lung cancer patients (55.0% [61/111] vs. 71.3% [72/101]; p = 0.014). Whereas the proportion of patients with complete remission was higher in patients without metastasis, the proportion of progressive disease was higher in patients with metastasis compared to those without metastasis (p < 0.001). Compared to other groups, non-lung cancer with metastasis group were less likely to have had ongoing treatment plan (p = 0.020). Regarding laboratory findings, there were no significant differences in white blood cell (WBC) count, lactate and procalcitonin levels among the four groups, while non-lung cancer patients without metastasis had lower C-reactive protein (CRP) levels compared with other cancer patients (p < 0.001). Indication for mechanical ventilation also did not differ among the groups (Fig. 1). The median value of sequential organ failure assessment (SOFA) score was highest in non-lung cancer without metastasis group, followed by non-lung cancer with metastasis group, lung cancer with metastasis group, and lung cancer without metastasis group (p = 0.014).

**Table 1:** Baseline characteristics of cancer patients who underwent intubation in the emergency department.

	Total (N = 212)	Non-lung cancer patients		Lung cancer patients^†^		p-value
		Without metastasis (n = 43)	With metastasis (n = 68)	Without metastasis (n = 21)	With metastasis (n = 80)	
**Age, years**	66 (56–74)	66 (58–75)	64 (55–73)	69 (63–78)	65 (55–74)	0.240
**Sex, male**	133 (62.7)	28 (65.1)	33 (48.5)	16 (76.2)	56 (70.0)	0.024
**Body mass index,** kg/m^2^	22.4 (20.1–24.6)	21.7 (19.6–24.7)	22.2 (19.2–24.6)	22.2 (21.0–25.0)	22.7 (20.7–24.3)	0.743
**Comorbidities**						
Diabetes mellitus	45 (21.2)	15 (34.9)	10 (14.7)	5 (23.8)	15 (18.8)	0.075
Hypertension	61 (28.8)	11 (25.6)	20 (29.4)	7 (33.3)	23 (28.8)	0.932
Chronic liver disease	17 (8.0)	9 (20.9)	4 (5.9)	0 (0)	4 (5.0)	0.005
Cardiovascular disease	15 (7.1)	2 (4.7)	5 (7.4)	2 (9.5)	6 (7.5)	0.895
Chronic lung disease	13 (6.1)	4 (9.3)	1 (1.5)	3 (14.3)	5 (6.3)	0.125
**Site of primary malignancy**						—
GI system	27 (12.7)	11 (25.6)	16 (23.5)			
HBP system	26 (12.3)	10 (23.3)	16 (23.5)			
GU system	19 (9.0)	7 (16.3)	12 (17.6)			
Others	39 (18.4)	15 (34.9)	24 (35.3)			
**Metastasis**						
**Number of metastasis**						
1	70 (33.0)	—	38 (55.8)	—	33 (41.2)	
2	48 (22.6)	—	22 (32.4)	—	26 (32.5)	
3	22 (10.4)	—	8 (11.8)	—	14 (17.5)	
4	7 (3.3)	—	0 (0)	—	7 (8.8)	
**Site of metastasis**						
Brain	39 (18.4)	—	5 (7.4)	—	34 (42.5)	
Thorax	81 (38.2)	—	28 (41.2)	—	53 (66.2)	
Abdomen	74 (34.9)	—	44 (64.7)	—	30 (37.5)	
Bone	49 (23.1)	—	17 (25.0)	—	32 (40.0)	
Others	17 (8.0)	—	11 (16.2)	—	6 (7.5)	
**Performance status**						0.545
<2	91 (42.9)	19 (44.2)	27 (39.7)	12 (57.1)	33 (41.3)	
≥2	121 (57.1)	24 (55.8)	41 (60.3)	9 (42.9)	47 (58.7)	
**Disease status**						<0.001
Complete remission	25 (11.8)	16 (37.2)	3 (4.5)	6 (28.6)	0 (0.0)	
New or first recurrence	36 (17.1)	12 (27.9)	10 (14.9)	5 (23.8)	9 (11.2)	
Partial response or stable disease	55 (26.1)	9 (20.9)	20 (29.9)	8 (38.1)	18 (22.5)	
Progressive disease	95 (45.0)	6 (14.0)	35 (51.5)	2 (9.5)	53 (66.2)	
**Ongoing treatment plan**	164 (77.4)	32 (74.4)	45 (66.2)	19 (90.5)	68 (85.0)	0.020
**Laboratory findings**						
White blood cell, mm^3^^*^	9.8 (4.4–15.8)	9.1 (5.8–13.4)	9.9 (1.9–18.3)	10.9 (1.2–15.2)	11.4 (5.1–16.6)	0.652
C-reactive protein, mg/L^*^	10.2 (3.9–21.0)	4.1 (0.2–10.1)	11.1 (4.7–22.1)	14.0 (8.7–24.5)	12.4 (7.0–24.8)	<0.001
Lactate, mmol/L^*^	4.4 (2.2–8.8)	5.2 (2.1–9.7)	5.2 (2.7–9.8)	3.5 (2.3–5.2)	4.2 (2.2–8.9)	0.292
Procalcitonin, ng/mL^*^	1.6 (0.4–18.4)	1.8 (0.3–14.6)	7.2 (0.9–31.0)	0.5 (0.2–1.6)	1.4 (0.5–10.9)	0.050
PF ratio^*^	255 (124–415)	276 (114–439)	302 (154–450)	235 (146–341)	223 (114–374)	0.324
**SOFA score**	5 (3–9)	7 (4–12)	6 (4–9)	4 (3–8)	5 (3–7)	0.014

Data are presented as number (%) or median (interquartile range).

GI, gastrointestinal; HBP, hepato-biliary-pancreatic; GU, genitourinary, SOFA, sequential organ failure assessment; PF, ratio of arterial oxygen partial pressure to fractional inspired oxygen.

^*^Missing data included as follows; white blood cell (n = 21), C-reactive protein (n = 21), lactate (n = 25), procalcitonin (n = 87), and PF ratio (n = 10).

^†^Eleven patients had small cell lung cancer.

### Comparison of clinical characteristics of cancer patients according to 28-day mortality

Of the 212 patients with malignancy who were intubated for mechanical ventilation in the ED, 58.0% of the patients (n = 123) died within 28 hospital days. As shown in Table 2, there were no significant differences in age, sex, BMI, comorbidities, site of primary malignancy, performance status, ongoing treatment plan, and WBC count between the patients who died and those who survived by the 28^th^ hospital day. Patients who died were more likely to have metastasis (p = 0.004) with higher frequency of abdominal metastasis (p = 0.005) and more progressive disease status (p = 0.020). In addition, patients who died were more likely to undergo cardiopulmonary resuscitation (CPR) (34.1% [42/123] vs. 6.7% [6/89], p < 0.001) and less likely to have respiratory failure without sepsis (38.2% [47/123] vs. 75.3% [67/89], p < 0.001) as an indication for mechanical ventilation, compared with those who survived. Patients who died also had a higher level of CRP (median 11.6 mg/L [interquartile ranges {IQR}, 6.6–22.8 mg/L] vs. 7.6 mg/L [IQR, 1.3–17.1, mg/L], p = 0.007), lactate (median 6.6 [IQR, 2.9–11.5 mmol/L] vs. 2.7 mmol/L [IQR, 2.0–5.8 mmoL/L], p < 0.001), procalcitonin (median 5.4 ng/mL [IQR, 0.7–24.4 ng/mL] vs. 0.9 ng/mL [IQR, 0.2–4.9 ng/mL], p = 0.008]). Whereas the ratio of partial pressure to fractional inspired oxygen was lower in patients who died than those who survived (median 226 [IQR, 97–398] vs. 297 [IQR, 169–436], p = 0.016), SOFA score was higher in patients who survived than those who died (median 7 [IQR, 4–10] vs. median 4 [IQR, 3–6], p < 0.001).

**Table 2:** Comparison of clinical characteristics of cancer patients according to 28-day mortality.

	Patients who survived (n = 89)	Patients who died (n = 123)	p-value
**Age, years**	65 (54–74)	66 (57–74)	0.752
**Sex, male**	58 (65.2)	75 (61.0)	0.632
**Body mass index,** kg/m^2^	22.5 (19.6–24.5)	22.3 (20.2–24.6)	0.990
**Comorbidities**			
Hypertension	25 (28.1)	36 (29.3)	0.973
Diabetes mellitus	22 (24.7)	23 (18.7)	0.375
Chronic liver disease	6 (6.7)	11 (8.9)	0.744
Chronic lung disease	5 (5.6)	8 (6.5)	>0.999
Cardiovascular disease	6 (6.7)	9 (7.3)	>0.999
**Site of primary malignancy**			
Lung^*^	37 (41.6)	64 (52.0)	0.172
GI system	4 (4.5)	15 (12.2)	0.090
HBP system	14 (15.7)	12 (9.8)	0.273
GU system	1 (1.9)	5 (7.0)	0.356
Others	24 (27.0)	15 (12.2)	0.010
**Any Metastasis**	52 (58.4)	96 (78.0)	0.004
**Multiple metastases** ^†^	26 (29.2)	51 (41.5)	0.005
**Site of metastasis**			
Brain	12 (13.5)	27 (22.0)	0.164
Thorax	30 (33.7)	51 (41.5)	0.315
Abdomen	21 (23.6)	53 (43.1)	0.005
Bone	18 (20.2)	31 (25.2)	0.494
Others	6 (6.7)	11 (8.9)	0.744
**Indication for mechanical ventilation**			
Respiratory failure without sepsis	67 (75.3)	47 (38.2)	<0.001
Sepsis-related respiratory failure	16 (18.0)	34 (27.6)	0.141
Cardiopulmonary resuscitation	6 (6.7)	42 (34.1)	<0.001
**Disease status**			0.020
No progressive disease	57 (64.0)	59 (48.0)	
Progressive disease	32 (36.0)	64 (52.0)	
**Performance status**			>0.999
<2	38 (42.7)	53 (43.1)	
≥2	51 (57.3)	70 (56.9)	
**Ongoing treatment plan**	70 (78.7)	94 (76.4)	0.829
**Laboratory findings** ^**††**^			
White blood cell, mm^3^	10.1 (5.7–13.7)	9.4 (1.8–17.2)	0.929
C-reactive protein, mg/L	7.6 (1.3–17.1)	11.6 (6.6–22.8)	0.007
Lactate, mmol/L	2.7 (2.0–5.8)	6.6 (2.9–11.5)	<0.001
Procalcitonin, ng/mL	0.9 (0.2–4.9)	5.4 (0.7–24.4)	0.008
PF ratio	297 (169–436)	226 (97–398)	0.016
**SOFA score**	4 (3–6)	7 (4–10)	<0.001

Data are presented as number (%) or median (interquartile range).

GI, gastrointestinal; HBP, hepato-biliary-pancreatic; GU, genitourinary; SOFA, sequential organ failure assessment; PF, ratio of arterial oxygen partial pressure to fractional inspired oxygen.

^*^Eleven patients including three who survived and eight who died had small cell lung cancer.

^†^Defined as two or more metastatic lesions.

^††^Missing data included as follows; white blood cell (n = 21), C-reactive protein (n = 21), lactate (n = 25), procalcitonin (n = 87), and PF ratio (n = 10).

### The impact of the coexistence of lung cancer and metastasis on 28-day mortality in cancer patients who were intubated in the ED

As shown in Table 3, 28-day mortality rates were 44.2% (19/43) in non-lung cancer patients without metastasis, 63.2% (43/68) in non-lung cancer patients with metastasis, 52.4% (11/21) in lung cancer patients without metastasis, and 66.2% (53/80) in lung cancer patients with metastasis. In both univariable and multivariable analyses, compared with non-lung cancer patients without metastasis, non-lung cancer patients with metastasis (unadjusted OR = 2.90, 95% confidence interval [CI] = 1.32–6.40; adjusted OR = 4.24, 95% CI = 1.32–13.65) and lung cancer patients with metastasis (unadjusted OR = 3.31, 95% CI = 1.53–7.17; adjusted OR = 7.17, 95% CI = 2.14–24.01) had significantly higher 28-day mortality, respectively. In comparison, whereas there was no significant increase of 28-day mortality in lung cancer patients with metastasis compared to non-lung cancer patients without metastasis in univariable analysis (p = 0.251), lung cancer patients with metastasis were 5.89 (95% CI = 1.48–23.36) times more likely to have higher 28-day mortality compared to non-lung cancer metastasis.

**Table 3:** Unadjusted and adjusted odds ratio for 28-day mortality in cancer patients who were intubated in the emergency department, stratified by the presence of lung cancer and metastasis.

	Non-lung cancer patients		Lung cancer patients		
	Without metastasis (n = 43)	With metastasis (n = 68)	Without metastasis (n = 21)	With metastasis (n = 80)	p-value
No. of dead patients	19 (44.2)	43 (63.2)	11 (52.4)	53 (66.2)	0.012
Unadjusted OR (95% CI)	Reference	2.90 (1.32–6.40)	1.86 (0.65–5.34)	3.31 (1.53–7.17)	—
Adjusted OR^*^ (95% CI)	Reference	4.24 (1.32–13.65)	5.89 (1.48–23.36)	7.17 (2.14–24.01)	—

OR, odds ratio; CI, confidence interval; BMI, body mass index; CPR, cardiopulmonary resuscitation; SOFA, sequential organ failure assessment.

^*^The clinical variables entered into the model included age, sex, BMI, chronic liver disease, chronic pulmonary disease, disease status, indication of intubation (respiratory failure without sepsis [reference] vs. sepsis-related respiratory failure or CPR), performance status, SOFA score, ongoing treatment plan, multiple metastases (defined as two or more metastatic lesions), and groups stratified by cancer type and the presence of metastasis (non-lung cancer without metastasis [reference] vs. non-lung cancer with metastasis, lung cancer without metastasis, or lung cancer with metastasis.

Regarding other clinical factors associated with 28-day mortality, compared to respiratory failure without sepsis, sepsis-related respiratory failure (unadjusted OR = 3.01, 95% CI = 1.50–6.11; adjusted OR = 2.60, 95% CI = 1.16–5.84), CPR (unadjusted OR = 9.98, 95% CI = 3.92–25.37; adjusted OR = 13.34, 95% CI = 4.45–39.95) and SOFA score (unadjusted OR = 1.14, 95% CI = 1.06-1.23; adjusted OR = 1.15, 95% CI = 1.05-1.26) were significantly associated with 28-day mortality in both univariable and multivariable analyses. Although progressive disease was associated with increased 28-day mortality in univariable analysis (unadjusted OR = 1.93, 95% CI = 1.10–3.38), this was not significant in adjusted model (p = 0.299) (Supplemental Table 1).

## Discussion

In the present study, we evaluated the clinical characteristics and treatment outcomes of lung cancer patients with metastasis who underwent mechanical ventilation in the ED and were admitted to the medical ICU. Among the included patients, all of whom had solid cancer, approximately one-half had lung cancer; of these, approximately 80% had metastatic disease. The most common indication for invasive mechanical ventilation in lung cancer patients with metastasis was respiratory failure without sepsis. The 28-day mortality in lung cancer patients with metastasis who underwent mechanical ventilation in the ED was approximately 66%. Lung cancer patients with metastasis were about 7.2 times more likely to die within 28 days compared with non-lung cancer patients without metastasis.

Lung cancer patients who develop acute illness requiring ICU admission often require intubation and mechanical ventilation. Previous studies showed that at least 40% of lung cancer patients who were admitted to the ICU required mechanical ventilation^7–15^. Unfortunately, the need for mechanical ventilation has been shown to be independently associated with ICU and hospital mortality^7–11,13,16,17^, and in most studies, fewer than one-half of lung cancer patients who received mechanical ventilation survived after hospitalization^7,8,11,12,14,18^. Unpredicted ICU admission is another factor associated with poor treatment outcomes in lung cancer patients, especially in those who were admitted from the ED^5^. Thus, it might be postulated that lung cancer patients who were intubated in the ED and admitted unexpectedly to the ICU for mechanical ventilation would have even worse treatment outcomes. However, there have been no comprehensive studies showing the natural courses of such patients at a critical juncture, i.e., supportive care for imminent death versus intensive care. With this view, our study provided informative data showing the natural courses of patients with serious medical conditions.

Another important finding in our study is that treatment outcomes in lung cancer patients without metastasis also were worse than non-lung cancer patients without metastasis. These results suggest that lung cancer patients might be more vulnerable to mechanical ventilation than other cancer patients, probably due to the involvement of cancer in the lung. Our results with the results from previous studies^10,13,16,19^ suggest that the decision whether to proceed with unplanned intubation and mechanical ventilation should be discreetly decided in lung cancer patients, particularly those with metastatic/progressive disease. However, since there were only 21 lung-cancer patients without metastasis, further studies with a larger volume of patients are needed to confirm this issue.

There have been conflicting reports regarding treatment outcomes of lung cancer patients who were treated in the ICU. Along with recent advances in critical care in general, ICU treatment outcomes in lung cancer patients reported from single-center studies showed a steadily improving trend^9–11,14,18,19^. In contrast, one study of 49,373 lung cancer patients admitted to the ICU from Surveillance, Epidemiology, and End Result–Medicare registry revealed that ICU outcomes in this population did not improve from 1992 to 2005^17^. Regarding factors associated with treatment outcomes, a recent prospective multinational study of 449 patients found that survival after ICU admission greatly differed according to performance status and recurrent or progressive cancer status. Patients with good performance status and non-recurrent/progressive disease were more likely to survive, and over one-third of hospital survivors received anti-cancer treatment after discharge^16^. Similar findings were reported from a single-center study in Korea^20^. Given that there has been a major paradigm shift in the treatment of advanced non-small-cell lung cancer^21^, patients with advanced lung cancer should not be excluded from ICU admission solely based on their initial disease stage. Rather, their performance status and future eligibility for preplanned anti-cancer treatment should be taken into account when making this decision. For patients with poor performance status and who are not candidates for anti-cancer therapy, early integration of palliative care would result in more frequent documentation of resuscitation preference and less-aggressive end-of-life care^22^. Our study suggests that this should be discussed before the patient develops a life-threatening medical condition requiring a visit to the ED with indications for mechanical ventilation, as thoughtful consideration and discussion is usually impossible at that time. In addition, not only these factors described above, but also accompanying factors such as types of cancer, the underlying comorbid illnesses in the acute organ dysfunction processes, should be taken into account in the decision of mechanical ventilation. In this study, compared to those with only respiratory failure, those with sepsis and underwent CPR had a higher risk of mortality. Furthermore, acute organ dysfunction process measured by SOFA score was an independent predictor associated with poor outcomes. From this perspective, we suggest that an emergent multidisciplinary approach comprising the intensivist, oncologist, and pulmonologist might be helpful to plan the optimal treatment for these patients.

This study has several limitations. First, our study was conducted at a single tertiary referral hospital, with a comprehensive cancer center and a specialized ICU for critically ill cancer patients. This may limit the generalizability of our findings. Second, our study design was retrospective. Third, information on treatment-limitation decisions after intubation, which might have influenced the mortality in cancer patients, is not provided in this study. Regardless of these limitations, our study included only patients with solid cancer who initiated mechanical ventilation in the ED, and provides valuable information on the prognosis of these patients according to cancer type and the presence of metastasis.

In conclusion, the outcome in lung cancer patients with metastasis who were intubated in the ED and admitted to the medical ICU for mechanical ventilation was significantly worse than non-lung cancer patients without metastasis. Sepsis-related respiratory failure and CPR over respiratory failure without sepsis and acute organ dysfunction process were significantly associated with an increased risk of mortality. Clinicians should be aware of these outcomes and, via a multidisciplinary approach with a consideration of types of cancer and the underlying comorbid illnesses in the acute organ dysfunction processes, discuss aggressive resuscitation versus end-of-life care with the patients and family in advance of an unexpected medical crisis.

## Methods

### Patients

Using the electronic medical record database, 212 consecutive patients with solid malignancy who were intubated in the ED and admitted to the ICU in Samsung Medical Center (a 1,979-bed referral hospital in Seoul, South Korea), between January 2014 and December 2016, were identified. Demographic and clinical data including age, sex, BMI, comorbidities, site of primary malignancy and the presence of distant metastasis, indication for mechanical ventilation, initial laboratory findings (WBC count, C-reactive protein, lactate, and procalcitonin), and 28-day mortality were collected from the review of the medical records. The Institutional Review Board of Samsung Medical Center approved this study and the need for written patient consent was waived due to the retrospective nature of the study (IRB no. 2017-09-066). This study was performed in accordance with relevant guidelines/regulations of our institutions.

### Definitions

Distant metastasis was defined as cancer involvement of distant organs, as seen on radiologic studies (computed tomography [CT], magnetic resonance imaging, or positron emission tomography-CT) with or without histologic confirmation. Indications for mechanical ventilation were categorized into respiratory failure without sepsis, sepsis-related respiratory failure, and CPR. Primary outcome was 28-day mortality, which was defined as all-cause mortality within 28 days from intubation. Malignancy of gastrointestinal system included malignancy of esophagus, stomach, small intestines, and large intestines. Malignancy of hepatobiliary-pancreas system included malignancy of liver, biliary system, gall bladder, and pancreas. Malignancy of genitourinary system included malignancy of urinary tract (kidney, ureter, and bladder) and genital system (ovary, uterine and uterine tube, cervix, vagina, and vulva in women and testis, prostate, and penis in men).

### Statistical Analyses

Continuous variables are reported as medians with IQR and categorical variables as numbers with percentages. Continuous variables were compared using the Mann–Whitney U-test, and categorical variables were compared using the chi-squared test with Yates’ continuity correction. Clinical characteristics and 28-day mortality were evaluated with stratification based on cancer type and the presence of metastasis as follows: non-lung cancer patients without metastasis, non-lung cancer patients with metastasis, lung cancer patients without metastasis, and lung cancer patients with metastasis. A multivariable logistic regression model was used to evaluate the OR of 28-day mortality in lung cancer patients with metastasis over non-lung cancer patients without metastasis. The initial clinical variables entered into the model were age, sex, BMI, chronic liver disease, chronic pulmonary disease, indication of intubation (respiratory failure without sepsis [reference] vs. sepsis-related respiratory failure or CPR), performance status, disease status, ongoing treatment plan, groups stratified by cancer type and the presence of metastasis, multiple metastases defined as two or more metastatic lesions, and SOFA score. To handle missing data in the regression model, the missing-indicator method, which is a popular and simple method to handle missing data in clinical research was used^23,24^. A two-sided p-value < 0.05 was considered to reflect statistical significance. All statistical analyses were performed using R Statistical Software (version 3.2.3) of the R Foundation for Statistical Computing, Vienna, Austria and Stata version 15.0 (Stata Corporation, College Station, TX, USA).

### Ethical approval and consent to participate

The Institutional Review Board of Samsung Medical Center approved this study and the need for written patient consent was waived due to the retrospective nature of the study (IRB no. 2017-09-066). This study was performed in accordance with relevant guidelines/regulations of our institutions.

## Supplementary information

Supplemental Table 1

## Data Availability

All data extracted in this study are included in this article.
