# Supplementary material for: Twenty-eight-day mortality in lung cancer patients with metastasis who initiated mechanical ventilation in the emergency department
Source: Sci Rep. 2019 Mar 20;9:4941. doi: 10.1038/s41598-019-39671-8 (PMC6427029; doi:10.1038/s41598-019-39671-8)
Supplement: Supplementary file 1 — Supplemental Table 1 [file 41598_2019_39671_MOESM1_ESM.docx]

**Supplemental Table 1.** Factors associated with 28-day mortality in patients who were intubated in emergency room

|  |  | Twenty-eight-day mortality | | |  |
| --- | --- | --- | --- | --- | --- |
|  | Univariable analysis | |  | Multivariable analysis^*^ |  |
|  | OR (95% CI) | | *p*-value | Adjusted OR (95% CI) | *p*-value |
| Age, year | 1.0 (0.98-1.03) | | 0.775 | 1.02 (0.99-1.05) | 0.289 |
| Sex |  | |  |  |  |
| Male | Reference | |  | Reference |  |
| Female | 1.20 (0.68-2.11) | | 0.533 | 1.17 (0.58-2.36) | 0.654 |
| BMI | 1.0 (0.92-1.08) | | 0.987 | 1.02 (0.99-1.12) | 0.730 |
| Performance status |  | |  |  |  |
| < 2 | Reference | |  | Reference |  |
| ≥ 2 | 0.98 (0.57-1.71) | | 0.955 | 0.63 (0.31-1.27) | 0.195 |
| Comorbidities |  | |  |  |  |
| Chronic liver disease | 1.36 (0.48-3.82) | | 0.561 | 1.66 (0.46-6.02) | 0.439 |
| Chronic pulmonary disease | 1.17 (0.337-3.70) | | 0.791 | 1.59 (0.36-7.15) | 0.543 |
| Ongoing treatment plan |  | |  |  |  |
| None | Reference | |  | Reference |  |
| Yes | 1.56 (0.86-2.72) | | 0.702 | 0.96 (0.41-2.28) | 0.932 |
| Disease status |  | |  |  |  |
| No progressive disease | Reference | |  | Reference |  |
| Progressive disease | 1.93 (1.10-3.38) | | 0.021 | 1.51 (0.69-3.27) | 0.299 |
| Indication of intubation |  | |  |  |  |
| Respiratory failure without sepsis | Reference | |  | Reference |  |
| Sepsis-related respiratory failure | 3.03 (1.50-6.11) | | 0.002 | 2.60 (1.16-5.84) | 0.020 |
| Cardiopulmonary resuscitation | 9.98 (3.92-25.37) | | < 0.001 | 13.34 (4.45-39.95) |  |
| Groups stratified by cancer type and the presence of metastasis |  | |  |  |  |
| Non-lung cancer without metastasis | Reference | |  | Reference |  |
| Non-lung cancer with metastasis | 2.90 (1.32-6.40) | | 0.008 | 4.24 (1.32-13.65) | 0.016 |
| Lung cancer without metastasis | 1.86 (0.65-5.34) | | 0.251 | 5.89 (1.48-23.36) | 0.012 |
| Lung cancer with metastasis | 3.21 (1.53-7.17) | | 0.002 | 7.17 (2.14-24.01) | 0.001 |
| Multiple metastases^†^ | 1.72 (0.96-3.07) | | 0.068 | 1.42 (0.62-3.24) | 0.404 |
| SOFA score | 1.14 (1.06-1.23) | | <0.001 | 1.15 (1.05-1.26) | 0.004 |

OR, odds ratio; CI, confidence interval; BMI, body mass index; CPR, cardiopulmonary resuscitation; SOFA, sequential organ failure assessment

^*^The clinical variables entered into the model included age, sex, BMI, chronic liver disease, chronic pulmonary disease, disease status, indication of intubation (respiratory failure without sepsis [reference] vs. sepsis-related respiratory failure or CPR), performance status, SOFA score, ongoing treatment plan, multiple metastases, and groups stratified by cancer type and the presence of metastasis (non-lung cancer without metastasis [reference] vs. non-lung cancer with metastasis, lung cancer without metastasis, or lung cancer with metastasis).

^†^Defined as two or more metastatic lesions.
